# Urban vegetation extraction from VHR (tri-)stereo imagery – a comparative study in two central European cities

**DOI:** 10.1080/22797254.2018.1431057

**Published:** 2018-02-06

**Authors:** Gyula Kothencz, Kerstin Kulessa, Aynabat Anyyeva, Stefan Lang

**Affiliations:** a Department of Geoinformatics - Z_GIS, University of Salzburg, Salzburg, Austria; b Spatial Services GmbH, Salzburg, Austria

**Keywords:** 3D extraction of urban vegetation, Pléiades (tri-)stereo imagery, semi-global matching, digital surface model, support vector machine

## Abstract

The present study proposes a workflow to extract vegetation height for urban areas from Pléiades stereo and tri-stereo satellite imagery. The workflow was applied on a stereo image pair for Szeged, Hungary and on tri-stereo imagery for Salzburg, Austria. Digital surface models (DSMs) of the study areas were computed using the semi-global matching algorithm. Normalised digital surface models (nDSMs) were then generated. Objects of vegetation and non-vegetation were delineated based on the spectral information of the multispectral images by applying multi-resolution segmentation and support vector machine classifier. Mean object height values were then computed from the overlaid pixels of the nDSMs and assigned to the objects. Finally, the delineated vegetation was classified into six vegetation height classes based on their assigned height values by using hierarchical classification. The vegetation discrimination resulted in very high accuracy, while the vegetation height extraction was moderately accurate. The results of the vegetation height extraction provided a vertical stratification of the vegetation in the two study areas which is readily applicable for decision support purposes. The elaborated workflow will contribute to a green monitoring and valuation strategy and provide input data for an urban green accessibility study.

## Introduction

Characterisation of the urban landscape can positively benefit from the vertical stratification of urban vegetation (Kumar, Pandey, & Jeyaseelan, 2012; Tenedorio, Encarnacao, Estanqueiro, & Rocha, 2006). Vegetation height in urban areas provides critical added value in many applications including utility planning, development or redevelopment of green infrastructure, green space management or biodiversity assessment and monitoring. This potential gain in information highlights the importance of three-dimensional (3D) representation of urban vegetation and poses high demand for 3D vegetation inventory and mapping (Kumar et al., 2012; Ren et al., 2014). Obtaining height information for vegetation in greater extent is challenging and has been rarely done for urban areas (Qayyum et al., 2015; Van Delm & Gulinck, 2011). Vegetation height can be obtained from field surveys, terrestrial and airborne laser scans and stereoscopic aerial or satellite images (Ullah, Dees, Datta, Adler, & Koch, 2017; Verma, Lamb, Reid, & Wilson, 2016). While field surveys are labour intensive and often based on estimations, laser scanning results in the highest spatial accuracy and precise 3D feature representation; still, laser scans are often beyond the budgets of most stakeholders (Larondelle & Strohbach, 2016; Verma et al., 2016). Very high resolution (VHR) stereoscopic satellite images are increasingly used as alternatives for laser scanning to compute DSMs of both urban and non-urban areas (Bachofer, 2016; Poli & Caravaggi, 2013). Several studies have experimented with extracting vegetation height from VHR satellite data. For example, Marsetič and Oštir (2010) assessed the potential of Ikonos stereo image pairs for vegetation height mapping for a 100 km^2^ study area in Slovenia. Van Delm and Gulinck (2011) also used Ikonos imagery for vegetation characterisation and volume estimation in urban and semi-urban complexes.

The agile acquisition capability of the Pléiades satellite system broadens the horizons of object height and 3D information extraction with near same time stereo or tri-stereo acquisition modes (Astrium, 2012). The synchronous stereo or tri-stereo acquisitions allow the production of DSMs, nDSMs and digital terrain models (DTMs) that are generally more accurate than those generated with multi-temporal imagery (Blonda, Marangi, Inglada, Mücher, & Lucas, 2013). For example, Luethje, Tiede, and Eisank (2017) extracted digital elevation model (DEM) in built-up areas from a Pléiades stereo scene. Fewer studies have applied the aforementioned capabilities of the Pléiades system for vegetation height modelling as yet. One of the examples used Pléiades stereo images to calculate vegetation height near power transmission poles in Malaysia (Qayyum et al., 2015). A literature search of the Scopus and the Web of Science databases for extraction of vegetation height for urban areas from Pléiades products did not reveal existing studies referenced in either of the two search engines (search terms: “Pléiades” and “vegetation height”; search date: 14 December 2017).

Consequently, this study addresses this research gap in presenting a workflow for stratifying vegetation height in urban settings from Pléiades stereo and tri-stereo imagery for urban planning and management purposes. The specific objectives of the study were:

1. to elaborate a vegetation height extraction workflow involving spectral and height information from Pléiades stereo and tri-stereo satellite imagery and
2. to demonstrate the application potential of the proposed workflow by extracting vegetation height classes for two central European urban settings: the cities of Szeged in Hungary and Salzburg in Austria.

Single methodological components of the workflow proposed are extensively discussed in other literature; in this study we try to merge these components to arrive at a consistent approach to derive vegetation height classes for urban planning purposes. The foundation of the workflow is based on two main components with several elements. First, the matching of stereo and tri-stereo image pairs of the Pléiades satellite system by using the semi-global matching (SGM) algorithm to produce point clouds for nDSMs that are used to extract vegetation height of the study areas. The second component is to classify and characterise the land cover based on the spectral and height properties of image segments by applying a Support Vector Machine (SVM) classifier.

Image matching is a precondition of object height extraction from (multiple-)stereo images. SGM is a robust and fast image matching algorithm, therefore it is frequently used by many authors (Gehrig, Eberli, & Meyer, 2009; Gehrke, Downey, Boehrer, & Fuchs, 2010). SGM is an area-based matching method that uses semi-global cost functions to match image pairs (Hirschmuller, 2008; Hirschmüller, 2005). The robustness and the accuracy of the algorithm have been highlighted in a number of studies. For example, SGM reduces outliers in low or non-textured areas while preserves edges and sharp object boundaries (Bethmann & Luhmann, 2015). Accuracy of point clouds generated from aerial photographs using SGM and airborne laser scanning have been systematically compared to estimate forest timber volume resulting in a remarkable agreement of the derived volumes (Ullah et al., 2017). Another study showed height value estimates of wood stands with slightly higher estimates for results obtained from SGM than that of airborne laser scanning (White, Stepper, Tompalski, Coops, & Wulder, 2015).

Image classification, based on spectral and other (e.g. spatio-temporal) features is the conversion of image data into semantic information, by manually or (semi-)automatically delineating objects of interest in the studied areas. Among the statistical, i.e. training-based classifiers, SVM is a robust, accurate and effective classifier for extracting land cover information from remotely sensed multispectral data (Gao & Liu, 2014; Nurul Iman Saiful, Asmala, & Burhanuddin Mohd, 2014). SVM is a supervised non-parametric learning algorithm that does not make assumptions about the frequency distribution of the data (Belgiu & Drăguţ, 2016; Mountrakis, Im, & Ogole, 2011). SVM is frequently used for both building and vegetation extraction with comforting accuracy in several studies. The extraction of building heights by using SVM classification of multispectral and height information of Pléiades satellite imagery resulted in medium to high accuracy in loosely built urban construct and in low accuracy in densely built areas (Bachofer, 2016). In a study presented by Cho, Malahlela, and Ramoelo (2015) SVM based tree species recognition yielded in 89.3% overall accuracy. Similarly, Zylshal, Yulianto, Nugroho, and Sofan (2016) extracted urban green space from Pléiades images using SVM classifier with a result of 86% overall accuracy.

Both evidences from the aforementioned studies and previous experiences inspired this work to elaborate a transferable urban vegetation extraction workflow, which is the subject of the present communication.

## Methods

### Study areas

**Figure 1.**
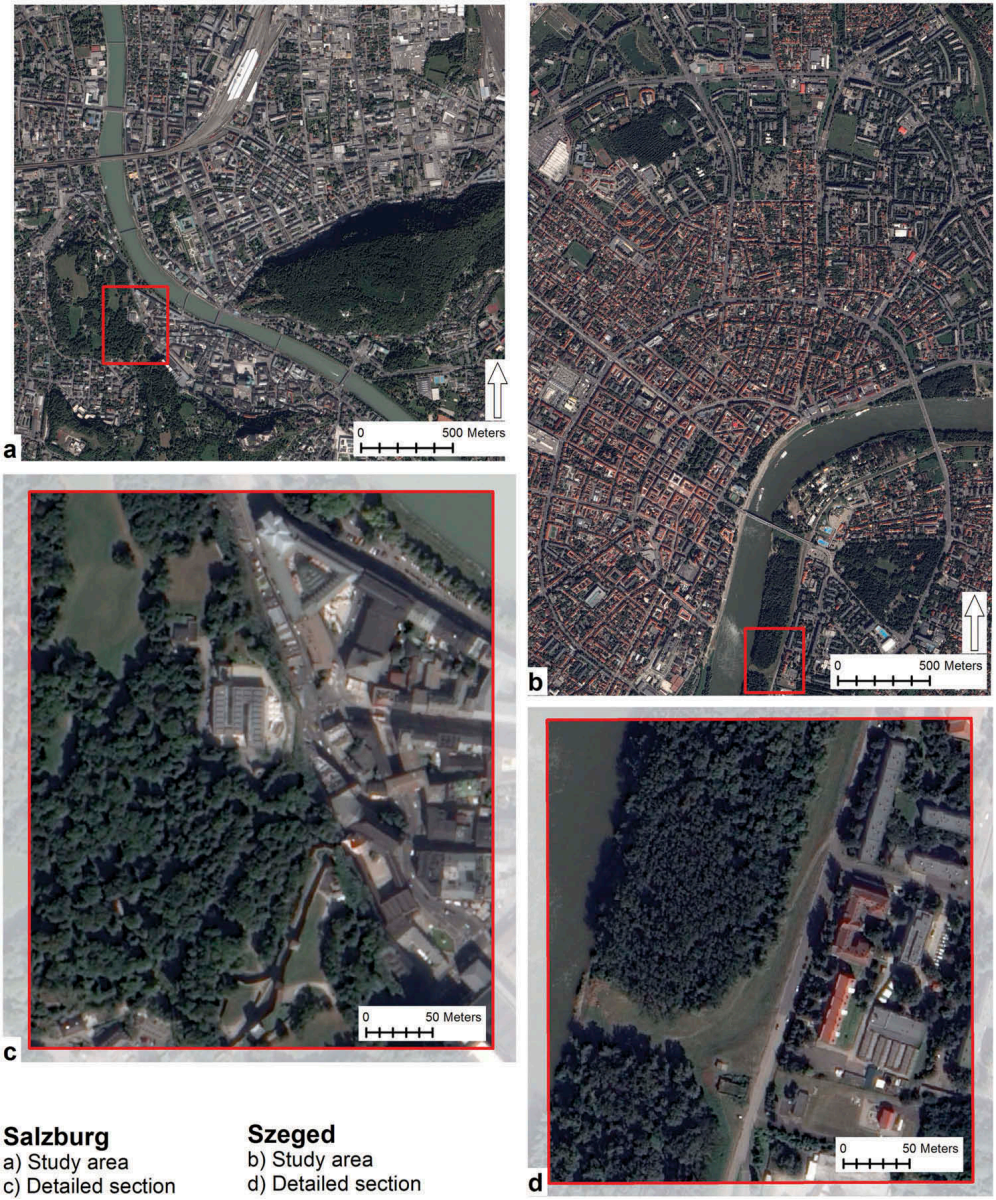
The two study areas: Salzburg (a) study area and (c) detailed section and Szeged (b) study area and (d) detailed section.

Located on the banks of the river Tisza, the City of Szeged is the capital of the South-East Hungary Euro Region and provides dwelling for 162,621 inhabitants (KSH, 2016). Comprising mainly the city centre and its surroundings, a 9.25 km^2^ (925.33 ha) area was clipped from the Szeged stereoscopic Pléiades image to serve as the Szeged study area (Figure 1(b, d)). The study area is characterised by a diverse urban landscape including buildings, sealed surfaces, unsealed vegetated surfaces and water surfaces.

The Austrian City of Salzburg sits astride the river Salzach. The settlement has 152,083 inhabitants and serves for the capital of Salzburg Province (Stadt Salzburg, 2016). Composed from the city centre and its surroundings, a 6.63 km^2^ (662.64 ha) area was clipped from the Pléiades image for the Salzburg study area (Figure 1(a, c)). Similarly to the Szeged study area, the Salzburg scene is equally diverse with similar land cover categories. The subset includes, the two major city hills, widely used for recreation. Towards the north-east of the study area, a mixed industrial/residential area is located that is bounded by the main train station and rail tracks to the north and the east.

Beyond their similarities the two study sites bear their own characteristics in terms of topography; while the Salzburg scene incorporates the two city hills, the Szeged area is flat.

### Data

#### Satellite images

Images from the Pléiades 1A satellite were used for both study areas. The constellation of the two satellites, Pléiades 1A and 1B, operating on the same orbit and phased 180° from each other (Astrium, 2012), enables a high image acquisition frequency and makes daily revisits over the same area possible. Multispectral Pléiades images cover four spectral bands: blue, green, red and near infrared with 2.0 m ground sampling. The resolution of the panchromatic band is 0.5 m. In stereo and tri-stereo acquisition mode two or respectively three images are recorded on the same target area at nearly the same time. As the satellite is able to rotate along its axis the same optical sensor can acquire images of the same target area from different viewing directions while moving along the orbit. One image is recorded in forward direction and the other one in backward direction. In tri-stereo mode an additional image, close-to-nadir, is recorded.

The images were recorded in stereo mode for Szeged, 120 km^2^ scene, and in tri-stereo mode for Salzburg, 168 km^2^. The acquisition parameters are detailed in Table 1.

**Table 1.** Acquisition parameters of the Salzburg image scene and the Szeged image scene.

**Salzburg**						
**Platform / Acquisition Mode**	**Acquisition Date**	**Acquisition Time**	**Viewing Angle (°) along track / across track**	**Incidence Angle (°) along track / across track**	**Sun azimuth / elevation (°)**	**Ground Sample Distance (m)**
PHR 1A / PX	1 September 2015	10:17:48 UTC	10.31 / 3.51	-11.83 / -1.27	160.46 / 49.24	0.72
PHR 1A / PX	1 September 2015	10:18:02 UTC	2.22 / 3.87	-3.16 / -3.66	160.83 / 49.29	0.70
PHR 1A / PX	1 September 2015	10:18:28 UTC	-11.53 / 4.48	11.66 / -7.73	160.83 / 49.29	0.73
**Szeged**						
**Platform / Acquisition Mode**	**Acquisition Date**	**Acquisition Time**	**Viewing Angle (°) along track / across track**	**Incidence Angle (°) along track / across track**	**Sun azimuth / elevation (°)**	**Ground Sample Distance (m)**
PHR 1A / PX	30 August 2014	09:51:41 UTC	10.22 / 9.92	-13.34 / -8.25	160.31 / 51.43	0.74
PHR 1A / PX	30 August 2014	09:52:27 UTC	-14.07 / 11.00	12.84 / -15.50	160.69 / 51.50	0.77

Both data sets were ordered as primary sensor products containing rational polynomial coefficients (RPC) information on each image in separate .xml files. RPC are required for photogrammetric calculations, since they provide information on the orientation of the images. For the further vegetation analysis pre-processing steps were carried out, including image calibration, orthorectification and pan-sharpening. The listed pre-processing steps were conducted for one image per study area, the one which was recorded closest to nadir. The top-of-atmosphere reflectance values were calculated. Afterwards, the images were orthorectified with a 5 m DTM for Szeged and a 10 m DTM for Salzburg. For pan-sharpening, the Gram–Schmidt method was used (Laben & Brower, 2000). The pre-processed images were clipped to the study areas.

#### Tree height ground reference

To validate the vertical accuracy of the vegetation extraction, additional data sets were used. For Szeged, a tree inventory of 42 trees of a city centre park, the Széchenyi tér, was available (DCLE, 2016). The data set was based on a terrestrial laser scanning performed in 2012. For Salzburg, a tree inventory that mostly covered the study area was sourced from the Salzburg municipality’s GIS database (Land Salzburg, 2011). The Salzburg tree inventory was based on atmospheric laser scanning performed in 2009.

#### Reference vegetation mask

For thematic accuracy assessment we used an automated pre-classification result using the Satellite Image Automatic Mapper (SIAM) software (A. Baraldi, Puzzolo, Blonda, Bruzzone, & Tarantino, 2006). SIAM^TM^ is an expert system (prior knowledge-based decision tree) for physical model-based vector quantisation in a multispectral feature space (Baraldi & Boschetti, 2012). SIAM was applied on TOA-calibrated and 8-bit coded reflectance values, and a vegetation mask was extracted by the implemented rule-based, four-bands feature space partitioning routine.

#### Ground control points

In both study areas, ground control points (GCPs) were used to enhance the accuracy of the image matching. GCPs for study areas were collected during field surveys, performed by earlier studies (DPGG, 2011; Kulessa & Lang, 2016), using real-time kinematic GPS technique.

#### Digital terrain models

DTMs of the two study areas were available for nDSM generation from earlier national mapping campaigns. The ground resolutions were 10 m in case of Salzburg and 5 m in case of Szeged.

#### Building height database of Szeged

This was used to calculate the threshold of the highest accepted height value of the Szeged point cloud within the SGM (Sümeghy, Gál, & Unger, 2011).

### Generation of the nDSMs

To ensure the comparability of the results, the same workflow was conducted for both study areas.

#### Image block orientation and triangulation

The panchromatic primary sensor products and their accompanying RPC files were inputted to a new image block. The RPC files defined the interior and exterior orientation of the block. Tie points were generated, then GCPs were imported to the stereo point measurement. Then triangulation (bundle block adjustment) was performed. The total root mean square error of the triangulation was 0.087 for Salzburg and 0.093 for Szeged.

#### Image matching

Prior to the matching process, lowest and highest accepted elevation limits of the prospective point clouds were recorded from the DTMs of the two areas, and from the building height database in case of Szeged, to reduce the number of points lying out of the set thresholds. Due to the hilly terrain, both the lowest and highest accepted elevation limits were obvious in Salzburg. Being flat, the highest points in the Szeged scene were the highest buildings of the city. For the stereo imagery of Szeged the single image pair (forward–backward direction) was inputted to the matching process. For the tri-stereo data of Salzburg three image pairs (forward–close-to-nadir; forward–backward and backward–close-to-nadir) were used. First, epipolar images were generated from the image pairs. Then elevation limits for the minimum and maximum accepted height values were set. The stereo matching was done by using the SGM algorithm. One point cloud was computed for Szeged and three for Salzburg. The computed point clouds contained points between the set height values, and the number of points outlying the set thresholds was greatly reduced by the algorithm. The point density of the resultant point clouds was 1.66 ppm^2^ for Salzburg and 3.71 ppm^2^ for the Szeged scene. The tree point clouds of Salzburg were merged to one. Both the Szeged and the merged Salzburg point clouds were then clipped to the respective study areas. Points remained beyond the accepted height values were removed from the clipped point clouds.

#### DSMs and nDSMs

**Figure 2.**
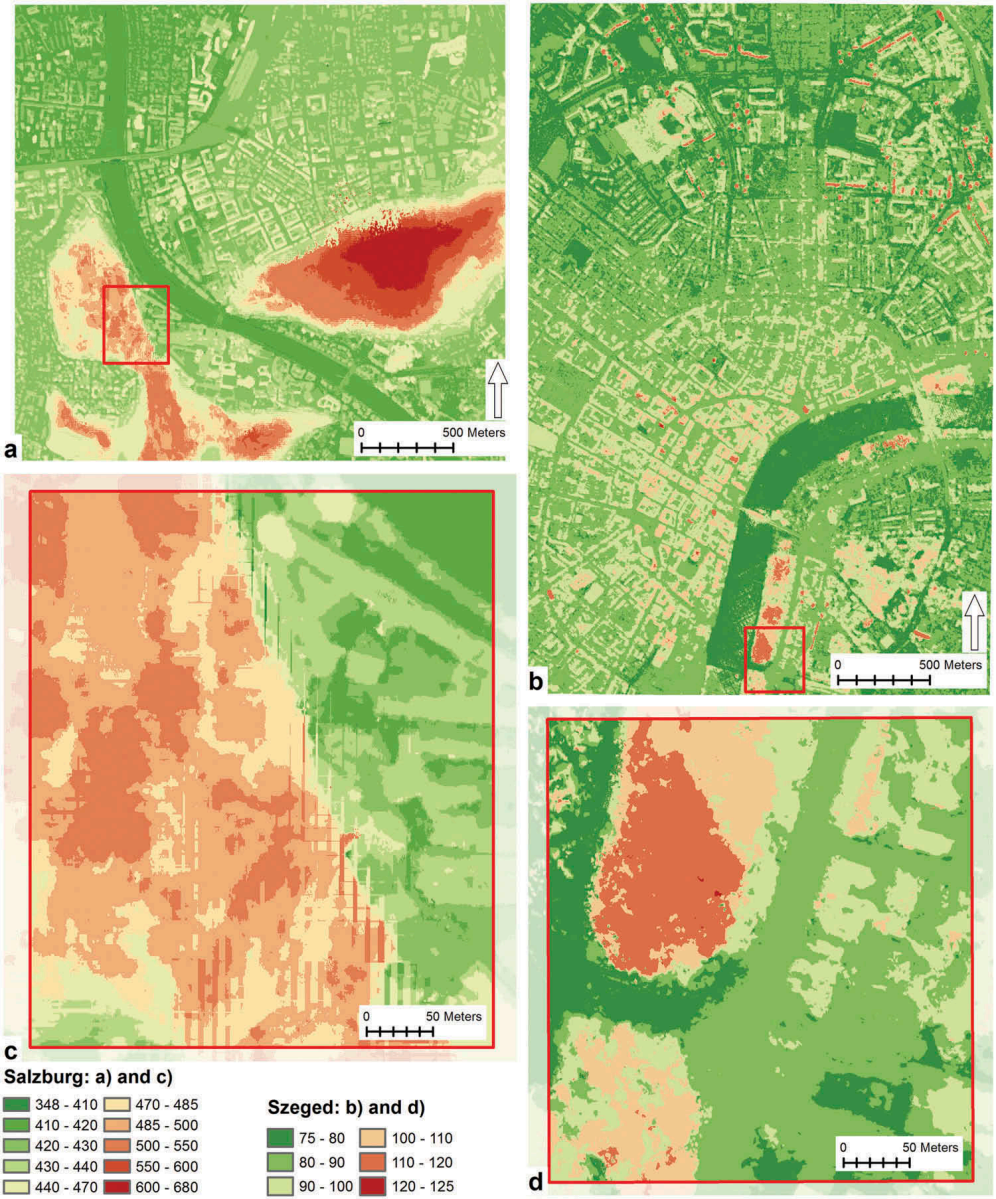
Digital surface models.

DSMs of the two study areas were computed from the two clipped then height-limited point clouds. The following parameters were used for gridding the point clouds. Horizontal sampling was set to 0.5 m. Adaptive triangulation was used; so, the triangulation allocated height values for void areas where there were no points found to assign elevation value to the corresponding cells. Minimum Z algorithm was used to set the cell values to the value of the lowest point in each grid cell to eliminate spikes caused by outlier point values. Due to the effective restriction of outlying points (described in the Image matching paragraph) no erroneous height values beyond the set limits propagated to the DSMs. The DSMs of the two study areas can be seen in Figure 2.

nDSMs were generated by subtracting the DTMs of the study areas from the DSMs.

### Outliers removal

**Figure 3.**
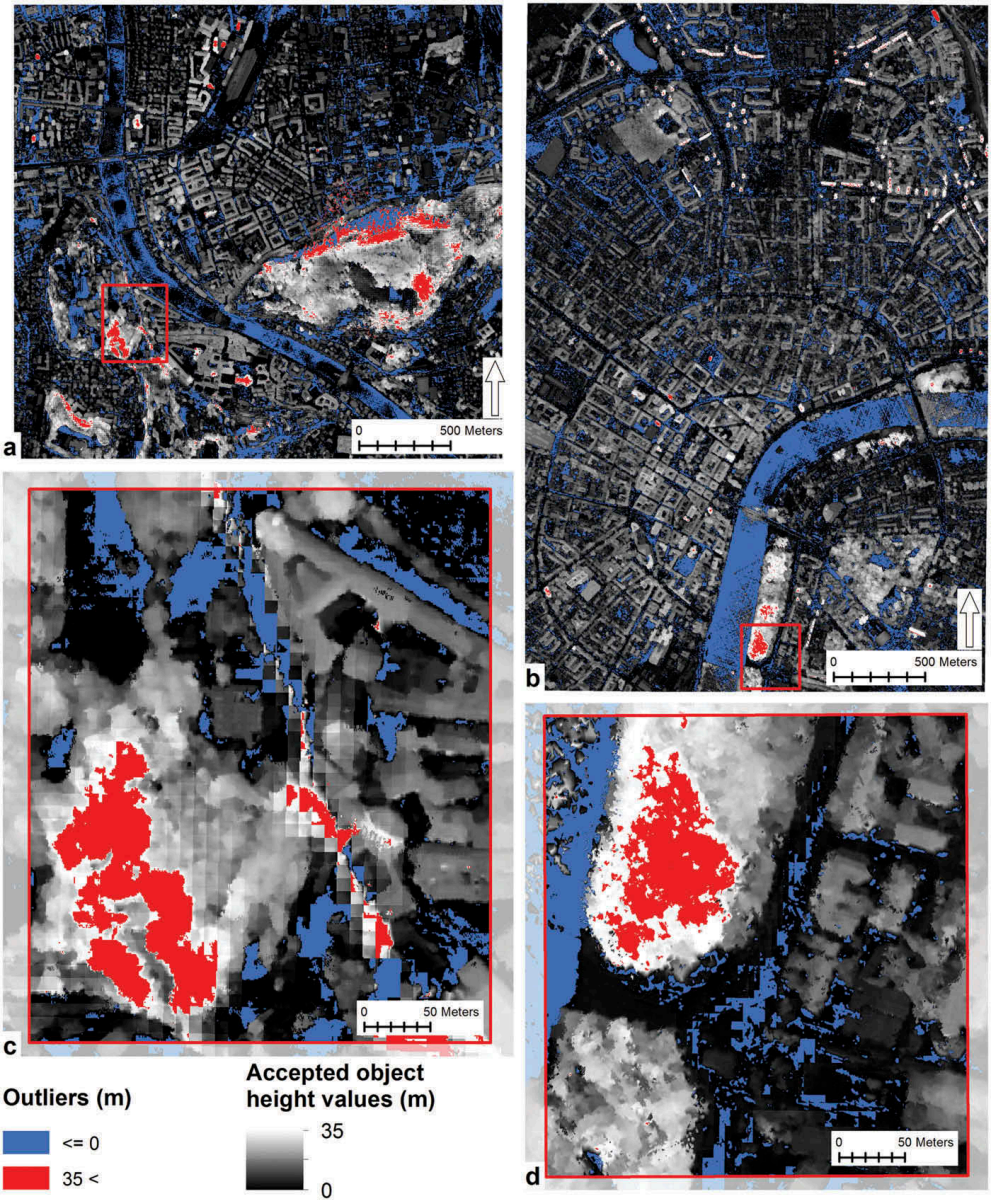
Outliers in the normalised digital surface models: Salzburg (a) study area and (c) detailed section and Szeged (b) study area and (d) detailed section.

In height models, generated by stereo matching, outliers can occur in low or non-textured surfaces, for example in shaded areas (Bethmann & Luhmann, 2015). Outliers can be caused, for example, by occlusions, transparent objects, slanted surfaces or radiometric artefacts like specular reflections (Baltsavias, Gruen, Eisenbeiss, Zhang, & Waser, 2008; Hirschmuller, 2008). Additionally, outliers can occur when the DSM is reduced to DTM or nDSM (Bethmann & Luhmann, 2015). The outliers result in extremely high or extremely low height values in the height model. Scrutinising the nDSMs of Salzburg and Szeged visually, outliers were found in shaded areas, in forests, on steep slopes and on water surfaces (Figure 3).

To comply with the purpose of the study, the correction of outliers was done in accordance to tree heights rather than heights of buildings. According to the reviewed literature the value for the maximum possible average tree heights was set to 35 m (Borhidi, 2003; Winkler, 2005). In nDSMs the minimum possible height value is 0. To remove the outliers from the nDSMs, a three-step process chain was executed in object-based image analysis software: (1) outlier detection by using pixel-based chessboard segmentation on a separate level and looping through each pixel in accordance to their height values; (2) correction of detected outliers by assigning them to the maximum value of vegetation height or to zero respectively; and (3) generation of a new outlier-free nDSM layer.

### Vegetation extraction

**Figure 4.**
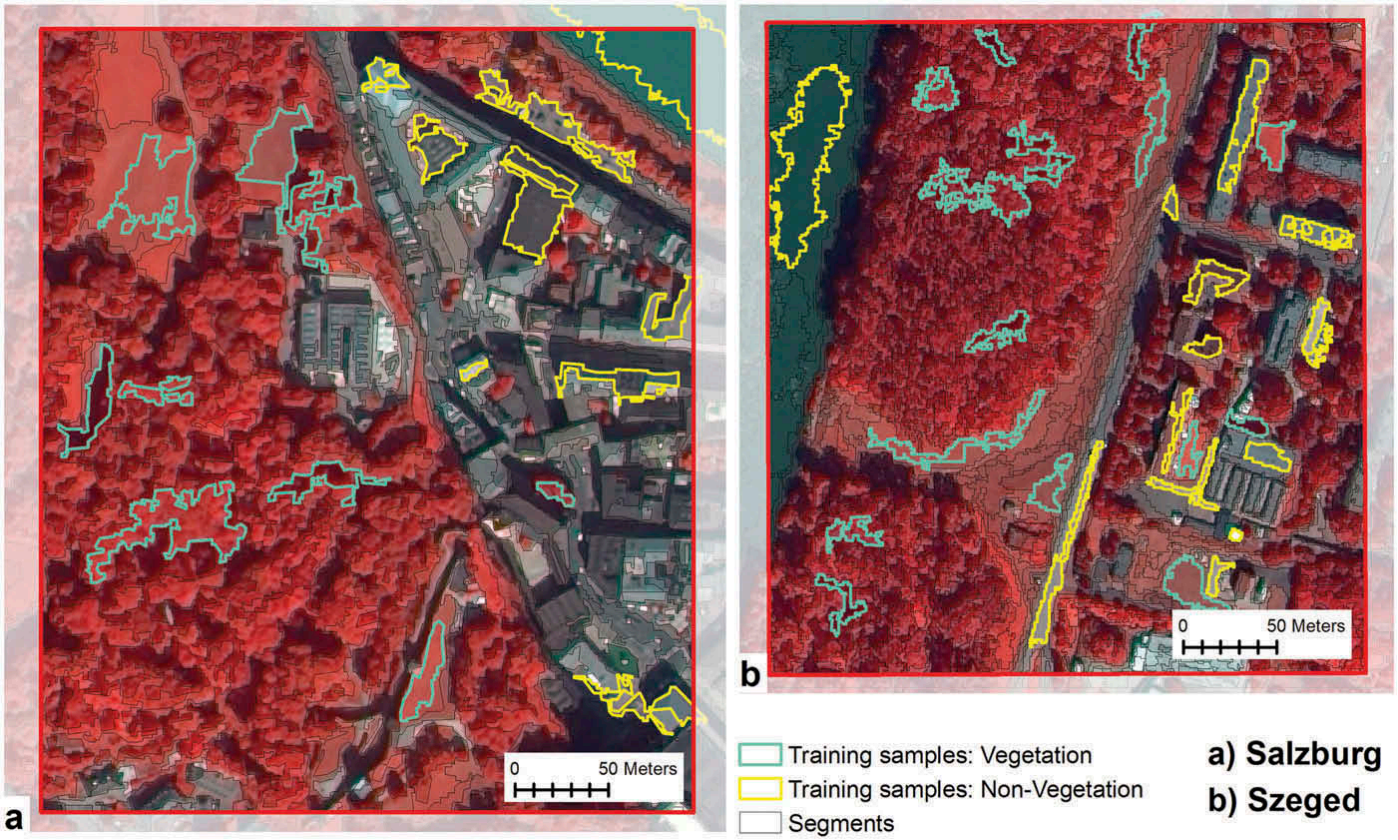
Results of the segmentation and selection of training samples.

The distribution of trees in urban areas is typically planned and aligned. However, the density and spatial concentrations of trees, and vegetation in general, vary and are oftentimes irregular, contributing therefore to structural complexity. Several studies argue that supervised classification methods may be effectively used to decompose urban complexity (Gao & Liu, 2014; Nurul Iman Saiful et al., 2014). To perform image segmentation of the multispectral image, the multi-resolution segmentation algorithm was used (Baatz & Schape, 2000). The algorithm was parameterised using the following values: the *scale parameter* was set to 200; layer weights for the four spectral bands and for the nDSM were set to 1. In order to emphasise the delineation of vegetated objects, we used the band arithmetic NDVI as a complementary layer. The *weight* for *shape* was set to 0.2 and the *compactness* was set to 0.7. The results of the segmentation for detailed sections of the study areas can be seen in Figure 4.

To differentiate vegetated surfaces from non-vegetated areas, a sample-based approach was used by applying the SVM classifier. Segments for training samples were selected for vegetation and non-vegetation classes based on false-colour composite rendering. The selection was based on visual perception of the false composite by choosing buildings, roads, sealed pavements, gravelled pavements, parking lots and water surfaces for non-vegetation, and trees, shrubs and grasslands for vegetation where they are obvious and easy to recognise. Figure 4 depicts the selection of training samples in detailed sections of the study areas.

The SVM algorithm was used to differentiate the multispectral image of the Salzburg study area into vegetation and non-vegetation classes. The classifier used the spectral signature of the four bands of the multispectral image to separate the two main land cover types. Thus, every object was assigned to one of the two categories and then obtained a height value. The algorithm was then transferred to the Szeged study area. The same process was used to separate vegetation and non-vegetation and to allocate height values for each object.

The algorithm could not classify objects of a steep, shaded slope of a hill in the Salzburg scene. By visual interpretation of the false composite and comparing it to the SIAM analysis, it was relatively straightforward to identify that the entire shaded area was covered with tree vegetation. Therefore, they were assigned to the vegetation class.

Objects of the vegetation class were vertically stratified into six vegetation height classes. Vegetation height classes were set as follows (in meters), with a fuzzified increasing and decreasing probability function at the margins of each class: grasslands (≤1), shrubs (>1 – ≤2.5), low trees (>2.5 – ≤10), medium high trees (>10 – ≤20), high trees (>20 – ≤30) and extremely high trees (>30). Then hierarchical classification was used to assign vegetation objects into one of the six categories. The results of the vertical stratification can be seen in the Results section (Figure 6).

**Figure 5.**
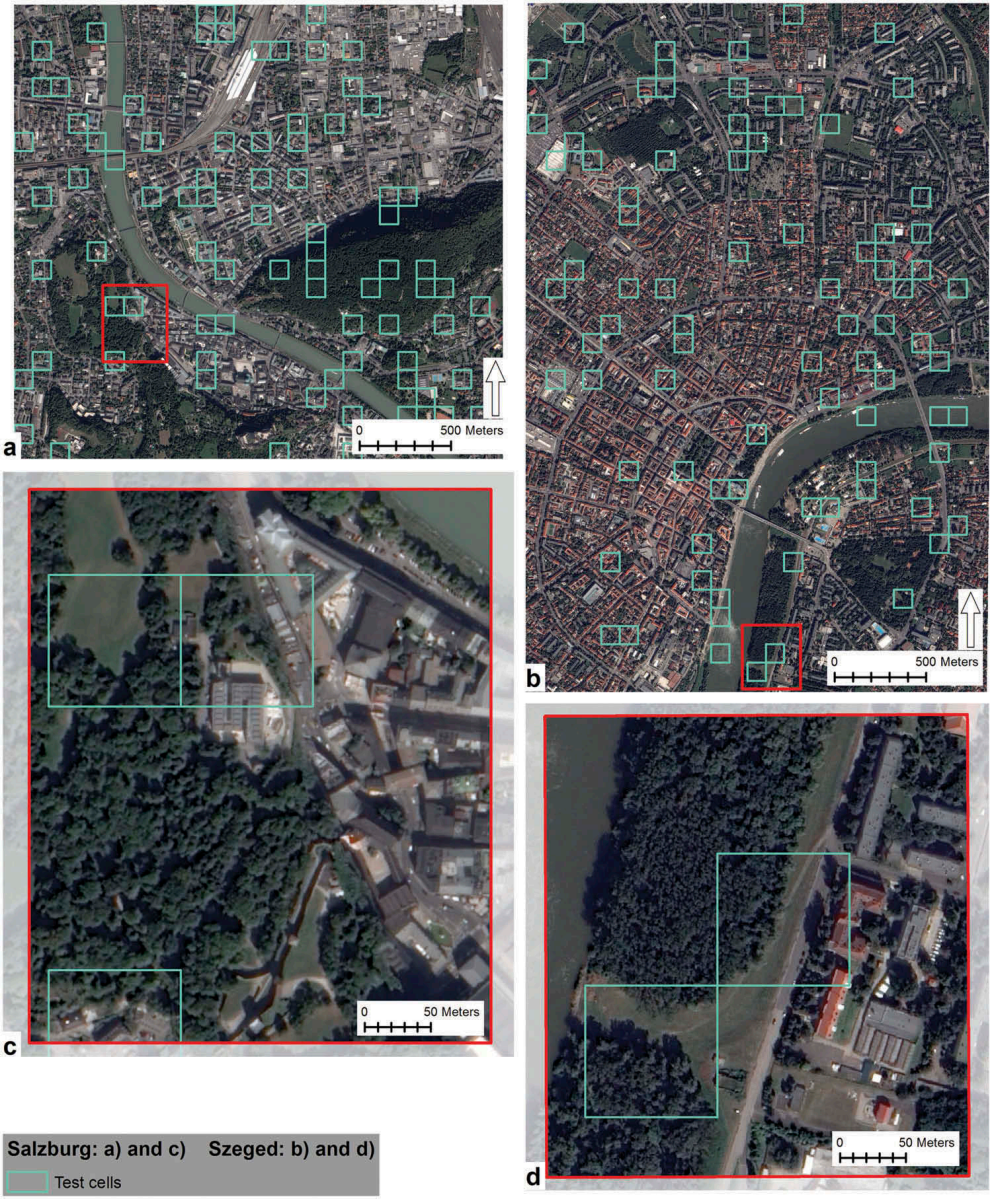
Test cells.

**Figure 6.**
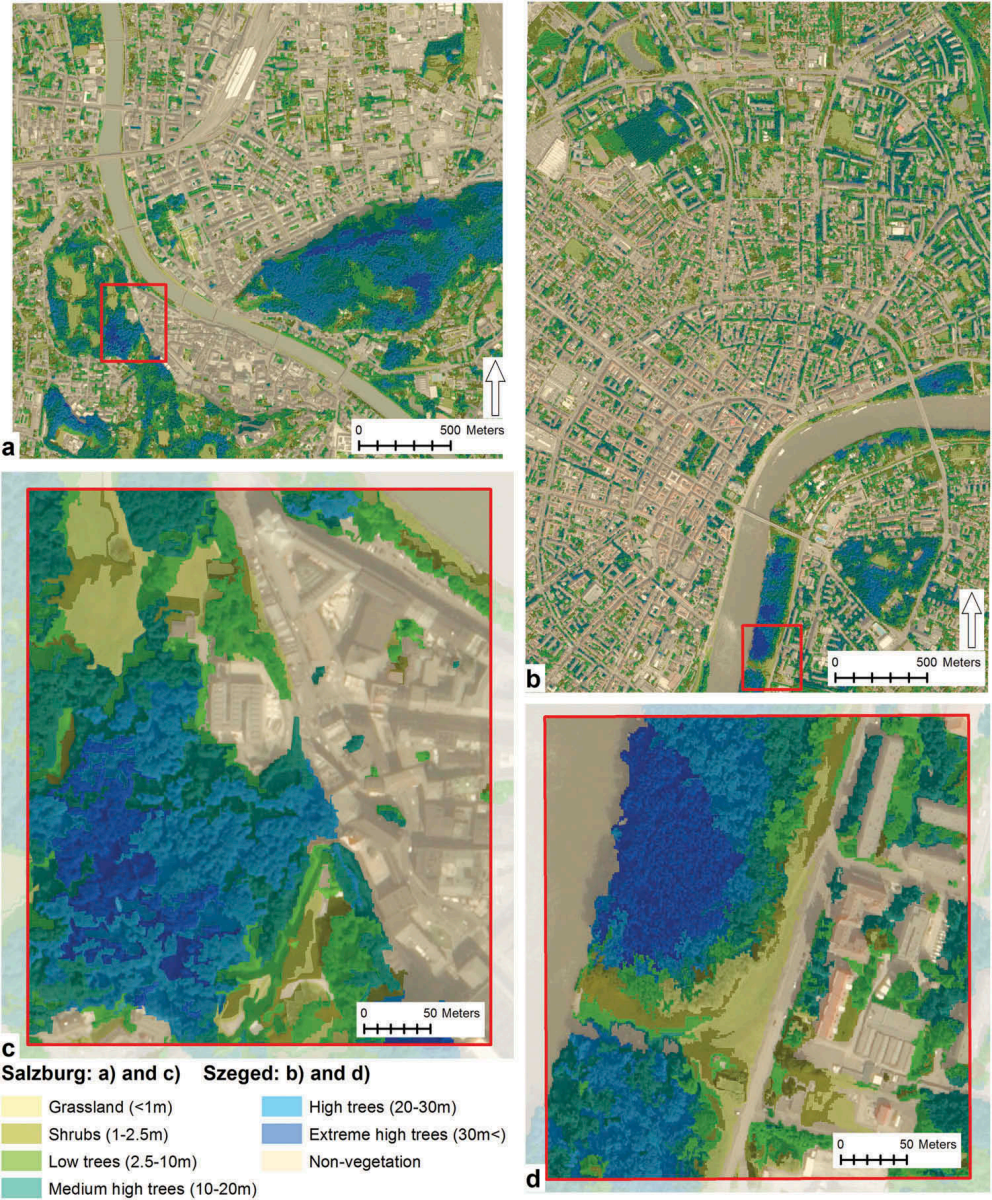
Vertical stratification of the extracted vegetation.

### Accuracy assessment

A thematic accuracy assessment was conducted. The AOI of the two study areas were tessellated in equally sized (100 m x 100 m) grid cells. Based on sampling size calculation (95% confidence level and 10% confidence interval), 85 cells for Salzburg and 87 for Szeged were randomly selected. Four cells in Salzburg and three in Szeged were removed as partly laying outside the AOI. Figure 5 shows the grid cells used for the accuracy assessment.

Two reference vegetation layers were used for validating the classification results. The first layer was generated by manually digitising the vegetation cover within each cell using the false-colour composite multispectral images for visual interpretation of the land cover. To create the second reference vegetation layer the 81 cells for Salzburg and the 84 cells for Szeged were intersected with a vegetation mask generated by a knowledge-based pre-classification routine as implemented in the SIAM software. Size of vegetated area per cell was computed for the two reference vegetation layers and for the SVM classification using zonal statistics. Metrics of descriptive statistics were then derived from the size of vegetation fractions per cell (Table 3). Standardised values of vegetation fractions delineated by the three methods were computed. For each cell, the proportion of vegetation fractions delineated by the SVM classifier was contrasted with the proportion of digitised vegetation and the proportion of SIAM vegetation mask (Table 4 and Table 5). The total area and proportion of vegetation for the 81 and 84 cells were finally calculated for all three vegetation layers and are shown in Table 6.

**Table 2.** Area and proportion of each delineated class to the study area.

	Salzburg		Szeged	
	Area of the class (ha)	Proportion of the class to the study area (%)	Area of the class (ha)	Proportion of the class to the study area (%)
Grass	48.01	7.25	53.08	5.74
Shrubs	41.23	6.22	62.36	6.74
Low trees	93.94	14.18	150.09	16.22
Medium high trees	62.42	9.42	91.98	9.94
High trees	39.74	6.00	14.40	1.56
Extremely high trees	6.32	0.95	2.64	0.28
Non-vegetation	370.98	55.99	550.79	59.52
Total	662.64	100.00	925.33	100.00

**Table 3.** Metrics of descriptive statistics – Salzburg and Szeged.

	Vegetated area – Salzburg			Vegetated area – Szeged		
	*Visual interpretation*	*SIAM*	*SVM*	*Visual interpretation*	*SIAM*	*SVM*
Mean (m^2^)	3798.56	3825.38	4256.94	3930.80	4398.43	4005.63
Standard Error (m^2^)	344.94	340.80	354.63	250.13	209.10	239.12
Median (m^2^)	3150.00	3076.00	3541.00	3606.50	4640.00	3997.50
Mode (m^2^)	10000.00	10000.00	10000.00	0.00	0.00	0.00
Standard Deviation (m^2^)	3104.48	3067.19	3191.63	2292.44	1916.40	2191.54
Kurtosis	-0.328721182	-0.454640228	-0.75072147	-0.174766275	0.358288313	-0.3602665
Skewness	0.894415668	0.766771926	0.595916437	0.428397186	-0.516029058	0.057756429
Range (m^2^)	10000.00	10000.00	10000.00	9972.00	9084.00	9472.00
Minimum (m^2^)	0.00	0.00	0.00	0.00	0.00	0.00
Maximum (m^2^)	10000.00	10000.00	10000.00	9972.00	9084.00	9472.00
Sum (m2)	307683.00	309856.00	344812.00	330187.00	369468.00	336473.00
Count (cells)	81	81	81	84	84	84

**Table 4.** Size and proportion of vegetation fractions per cell, standardised values, and deviation of proportion of the size of the SVM delineated vegetation fractions from the reference layers; ten randomly selected cells – Salzburgand Szeged.

	Reference layer 1.:Visual interpretation			Reference layer 2.: SIAM				SVM				
Cell ID	1. Vegetated area (m^2^)*	2. Vegetated area (%)**	3. Standardised values (Z-score)	4. Vegetated area (m^2^)*	5. Vegetated area (%)**	6. Standardised values (Z-score)	7. Deviation from the visual iterpretation (%)(2. - 5.)	8. Vegetated area (m^2^)*	9. Vegetated area (%)**	10. Standardised values (Z-score)	11. Deviation from the visual iterpretation (%)(2. - 9.)	12. Deviation from the SIAM vegetation mask (%)(5. - 9.)
99	40	0.4	-1.21069	44	0.44	-1.23285	-0.04	51	0.51	-1.3178	-0.11	-0.07
143	4529	45.29	0.235287	4740	47.4	0.298193	-2.11	5543	55.43	0.402948	-10.14	-8.03
189	5023	50.23	0.394412	5324	53.24	0.488595	-3.01	5988	59.88	0.542375	-9.65	-6.64
241	1734	17.34	-0.66502	1400	14	-0.79075	3.34	1519	15.19	-0.85785	2.15	-1.19
285	3552	35.52	-0.07942	4172	41.72	0.113008	-6.2	4765	47.65	0.159185	-12.13	-5.93
436	9893	98.93	1.963112	8204	82.04	1.427564	16.89	9994	99.94	1.797531	-1.01	-17.9
517	0	0	-1.22357	0	0	-1.24719	0	0	0	-1.33378	0	0
614	3196	31.96	-0.19409	4372	43.72	0.178214	-11.76	4606	46.06	0.109368	-14.1	-2.34
667	3642	36.42	-0.05043	3684	36.84	-0.0461	-0.42	3901	39.01	-0.11152	-2.59	-2.17
716	5636	56.36	0.591868	7028	70.28	1.044152	-13.92	5924	59.24	0.522322	-2.88	11.04

^a^Area of vegetation cover within each cell in square meters (m^2^). Area of each cell: 10,000 m^2^ ^b^Area of vegetation cover within each cell in percentage (%). Area of each cell: 10,000 m^2^ = 100%

### Validation of the vegetation heights

Extracted vegetation heights were validated against the tree cadastres available for the study areas. Three circumstances complicated the process. First, neither the horizontal ground sampling nor the object heights extracted from satellite images allow high enough accuracy for point-to-point ground referencing. Second, the locations of several reference points in the tree height cadastres were inaccurate. Third, the Szeged tree heights were acquired in continuous values which made the continuous segment heights and the reference data easily comparable. The Salzburg tree cadastre contained only discrete values, the upper thresholds of tree height classes, in 5-m intervals, to which particular trees belonged. To overcome the first two difficulties, horizontal buffer thresholds around the ground reference points and vertical thresholds on the delineated objects heights were applied. In Szeged, there were 42 trees available in the tree cadastre. In Salzburg, 85 trees were selected with a random selection generator. The number was chosen to equal the statistically significant number of 100 m by 100 m grid cells. For Salzburg, the following steps were done. Attributes of segments within 4 m search radius of the ground reference points were spatially joined to the attribute record of the point. In the joined table, delineated objects whose heights were within 2 m vertical threshold of the 5-m height interval of which the respective reference tree belonged to were marked as accurate estimations. A frequency table then quantified the correct height estimations. Objects heights in Szeged were compared to the continuous reference heights as follows. Attributes of segments within 4 m search radius of the ground reference point were spatially joined to the attribute record of the point. In the joined table, segments whose heights were within 4 m vertical threshold of the height of the respective reference tree were marked as accurate estimations. Records of the joined table were exported to a frequency table which quantified the accurate vertical estimations. For Szeged the interval-based validation procedure of Salzburg was also performed after classifying the continuous reference data in the vertical intervals of the Salzburg tree cadastre. Results of the vegetation height validation are described in the Results section.

## Results

### Results of vertical stratification of the extracted vegetation and site comparison

To demonstrate the application potentials of the proposed workflow, the extracted vegetation was classified based on their object heights and spectral information and results were plotted in Figure 6.

The area standardised proportion of the classified vegetation was similar in the two study areas. According to the results presented in Table 2 the differences were the following. The proportion of grasslands was greater in Salzburg by 1.51%. The proportion of shrubs was larger in Szeged by 0.52%. The proportion of areas vegetated with low and medium high trees was greater in the Szeged scene by 2.04% and 0.52%, respectively. The proportion of high trees was larger in Salzburg, accounting for 4.44%. The difference in the proportion of extreme high trees was 0.67% for Salzburg. And finally, the proportion of non-vegetated area was greater in Szeged by 3.53%.

### Results of the accuracy assessment

The digitised vegetation layer was considered as benchmark due to the precise visual interpretation of the land cover. Metrics of descriptive statistics derived from the size of vegetation fractions per cell resulted in major similarity between the visually interpreted and the SIAM vegetation layers in Salzburg (Table 3). For example, the means (visual interpretation: 3798.56; SIAM: 3825.38) and the standard deviations (visual interpretation: 3104.48; SIAM: 3067.19) showed only minor difference. Descriptive metrics of the SVM delineation were also highly comparable to those of the two reference layers (Table 3). In Szeged, descriptive statistics revealed that figures of the SVM delineation were near identical to those of the visual interpretation (Table 3). For example, means were 3930.80 for the visual interpretation and 4005.63 for the SVM, while the standard deviation was 2292.44 for the visual interpretation and 2191.54 for the SVM. Descriptive metrics of the Szeged SIAM vegetation mask differed in a small extent from those of the visual interpretation and the SVM (Table 3).

Differences in size of vegetation fractions in individual cells were more apparent for the three delineation types. Table 4 and Table 5 show the size, the proportion and the standardised values for 10 randomly selected cells along with the deviation of the proportion of the size of the SVM delineated vegetation fractions from those of the two reference layers.

**Table 5.** Size and proportion of vegetation fractions per cell, standardised values, and deviation of proportion of the size of the SVM delineated vegetation fractions from the reference layers; Ten randomly selected cells – Szeged.

	Reference layer 1.:Visual interpretation			Reference layer 2.: SIAM				SVM				
Cell ID	1. Vegetated area (m^2^)*	2. Vegetated area (%)**	3. Standardised values (Z-score)	4. Vegetated area (m^2^)*	5. Vegetated area (%)**	6. Standardised values (Z-score)	7. Deviation from the visual iterpretation (%)(2. - 5.)	8. Vegetated area (m^2^)*	9. Vegetated area (%)**	10. Standardised values (Z-score)	11. Deviation from the visual iterpretation (%)(2. - 9.)	12. Deviation from the SIAM vegetation mask (%)(5. - 9.)
87	6412	64.12	1.082339	6160	61.6	0.919209	2.52	6851	68.51	1.298344	-4.39	-6.91
106	5899	58.99	0.858561	6644	66.44	1.171766	-7.45	6673	66.73	1.217122	-7.74	-0.29
384	3218	32.18	-0.31093	5092	50.92	0.361914	-18.74	3818	38.18	-0.08562	-6	12.74
394	3710	37.1	-0.09632	4304	43.04	-0.04927	-5.94	3724	37.24	-0.12851	-0.14	5.8
544	4278	42.78	0.151455	6060	60.6	0.867028	-17.82	4762	47.62	0.345132	-4.84	12.98
549	804	8.04	-1.36396	364	3.64	-2.10521	4.4	233	2.33	-1.72145	5.71	1.31
551	870	8.7	-1.33517	1400	14	-1.56462	-5.3	678	6.78	-1.5184	1.92	7.22
674	5464	54.64	0.668807	5364	53.64	0.503847	1	5127	51.27	0.511682	3.37	2.37
812	2616	26.16	-0.57354	4656	46.56	0.134404	-20.4	2983	29.83	-0.46663	-3.67	16.73
822	8048	80.48	1.795988	6376	63.76	1.03192	16.72	7174	71.74	1.445729	8.74	-7.98

* Area of vegetation cover within each cell in square meters (m^2^). Area of each cell: 10000 m^2^ ** Area of vegetation cover within each cell in percentage (%). Area of each cell: 10000 m^2^ = 100%

**Table 6.** Overall results.

	Salzburg			Szeged		
	Visual interpretation	SIAM	SVM	Visual interpretation	SIAM	SVM
Count (cells)	81	81	81	84	84	84
Total area (ha)	81.00	81.00	81.00	84.00	84.00	84.00
Vegetated area (ha)	30.77	30.99	34.48	33.02	36.95	33.65
Vegetated area (%)	37.99	38.25	42.57	39.31	43.98	40.06

Overall results for the entire set of test cells showed significant similarities both in the area and proportion of vegetation delineated by the three methodologies. In Salzburg, the vegetation delineated by the SVM classifier was only 3.71 ha (4.58%) larger than the digitised vegetation cover and 3.49 ha (4.32%) larger than the SIAM vegetation mask. Overall figures of the visual interpretation and the SIAM layer were nearly identical in Salzburg. In Szeged, the size and proportion of the vegetation, delineated by the SVM classifier, were nearly identical to the metrics of the digitised reference layer. The SVM delineated merely 0.63 ha (0.75%) larger vegetation cover than the visual interpretation did. The SIAM delineation resulted in a minor over classification compared to the digitised vegetation layer. Therefore, the SVM results showed more pronounced but still minor difference in size (−3.3 ha) and proportion (−3.92%) from figures of the SIAM vegetation mask. Overall results of the accuracy assessment can be seen in Table 6.

### Results of the vegetation height validation

In Salzburg, 49 segment heights were within 2 m vertical threshold of the height interval of the respective tree out of the 85 reference points. This result accounts for 57.65% vertical accuracy. In Szeged, the quantitative comparison with the continuous reference data showed that 25 segment heights were within 4 m vertical threshold of the height of the respective reference point. The interval-based comparison resulted in 26 segments whose object heights were within 2 m vertical threshold of the height class of the respective reference tree. Considering the 42 available reference points, the comparison of continuous data sets resulted in 59.52% accurate height delineations, while the comparison of interval data showed 61.90% accuracy.

## Discussion

Vegetation height inventories in urban areas are essential to support informed decisions. Producing such databases is labour- and cost-intensive; therefore, city scale vegetation height inventories are hardly available. Pléiades imagery is widely accessible and raw data costs are relatively low compared to customised LIDAR acquisitions. This study has elaborated a workflow that extracts vegetation height in urban areas from Pléiades stereo and tri-stereo images. The accuracy assessment resulted in highly precise vegetation delineation for both study areas. In Salzburg, the vegetated area delineated by the SVM classifier deviated less than 5% from that in the two reference layers. In Szeged, the area of the SVM-delineated vegetation differed less than 1% from the area of the digitised vegetation. The difference between the SVM delineated vegetation and the SIAM vegetation mask was marginally larger (-3.92%). Since the area of the SVM delineated vegetation was nearly identical with that in the benchmark digitised vegetation cover in Szeged, here the SVM classifier performed better than the SIAM did. This is attributable to the slightly over classified SIAM layer. The minor over classification was an effect of different parameterisation of the SIAM that was necessary to be done due to the contrasting environmental settings of the two cities which is especially pronounced at the end of the summer when both the Szeged and Salzburg images were recorded. Due to significantly less summer rain in Szeged, the grasslands in August and September are rather dry in contrast to those in Salzburg. SIAM settings remained unaltered after the Salzburg classification assigned dry, shadowed grasslands of Szeged to the non-vegetation category. To include these areas in the vegetation class, the spectral category in SIAM “Dark Built-up and Barren land” was added to the vegetation with the argument that this is the dry, shadowed vegetation.

Quantitative comparison of extracted vegetation heights with the reference data showed moderate reliability. In Salzburg only comparison to interval data was possible eventuating in 57.65% correct height estimations. In Szeged the comparison of continuous height values resulted in 59.52% accurate height estimations. Interval-based comparison showed a slightly better estimation rate (61.90%). There is a possible argument to explain the moderate vertical accuracy. Height models computed even from VHR imagery cannot achieve high enough horizontal and vertical accuracy that a 2- or 4-m test point search radius would tolerate in areas where relatively large, but sudden changes in height occur. And drastically contrasting height values are frequent in urban space. The medium vertical accuracy is confirmed by Van Delm and Gulinck (2011) who found it relatively less demanding to separate grassland from high vegetation and argued that it is challenging to separate elements of high vegetation.

Despite the capability of SGM in resolving textural problems (Hirschmuller, 2008), the algorithm was not able to generate data points or computed mostly outliers with erroneous vertical values on dark areas with low or no texture, and on homogeneous surfaces. Similarly, erroneous height values were measured in wooded areas with larger stands of extremely high trees. Beyond these, inaccurate height values were detected on steep slopes with high trees and on water surfaces. Baltsavias et al. (2008) and Bethmann and Luhmann (2015) noted the same weaknesses of image matching algorithms. These experiences suggest that one needs to be aware of the limitations of satellite images and image matching algorithms when extracting vegetation height in such challenging surfaces. These observations are in line with the findings of earlier studies that derived 3D information from satellite images. For example, Luethje et al. (2017) noted that accuracy of DEM computed from Pléiades stereo images was limited at slopes covered with high vegetation. Bachofer (2016) concluded that object height extraction from WorldView-3 images is challenging in areas with complex topography and often requires manual post-processing.

The method elaborated in this study has been particularly designed and scaled to Pléiades products. Horizontal and vertical heterogeneity of the fine scale offered by Pléiades images may not be detected using coarse spatial resolution satellite images (Kitron et al., 2006). When the classification is transferred to other Pléiades images new parameter settings are required, and manual selection of training samples needs to be conducted for every new image. Even when effective in singular studies, SVM classifiers – as other sample-based learning algorithms – face general limitations in transparency and transferability, because samples need to be collected individually, therefore SVM classifiers are sensitive to segment selection (Belgiu & Drăguţ, 2016).

To eliminate the manual interference and reduce the dependency on training sample selection, further developments towards rule-based automatisation are foreseen. The workflow is designed in such a way that single elements can be replaced by others (e.g. the sample-based differentiation between vegetation and non-vegetation segments could be replaced by knowledge-based routines), without changing the overall purpose. New development will combine elements of the process chain, reported in this study, and the rule-based reference classification as described above.

The application potentials of the workflow and its generated results are already foreseen. First, an urban green monitoring and evaluation strategy will be developed by the authors of the present communication for which the workflow elaborated here will provide methodological input. This will significantly enhance the overall performance of regular green monitoring activities, as planned in an interval of 5 years in the city of Salzburg with a recent update round based on the tri-stereo Pléiades data acquired in 2015 and used by this study. Hitherto, analyses had been performed on two-dimensional remote-sensing data only, and it is now envisaged to include the experience of the present study in a more differentiated green evaluation assessment. Second, an analysis of personal availability of Szeged’s green environment will be performed. The classification results obtained through the developed workflow elaborated in the recent communication will be used as data input for the identification of Szeged’s green structures which would be the subject of the availability analysis. Finally, the authors’ recommendation for decision support is the following. The accuracy of vegetation heights derived from Pléiades images can at this stage hardly compete with laser scanning. Pléiades images on the other hand can be updated regularly on lower cost than series of LiDAR acquisitions could be. And if the topography of the study area is not so demanding that the resolution of the panchromatic band is sufficient for a desired application, they can provide good alternatives to LiDAR data or field surveys to estimate vegetation height in urban areas.

## Conclusion

This study has presented a 3D vegetation extraction workflow that used (tri-)stereo Pléiades imagery for vertical vegetation stratification. SGM and SVM were the main cornerstones of the methodology that was applied for a stereo scene in Szeged, Hungary and a tri-stereo scene in Salzburg, Austria. Extracted vegetation was assigned to six categories according to their height values in the nDSM. Non-vegetated surfaces formed another, seventh category. The results of the study were the following. The classification accuracy of vegetation cover and non-vegetated surfaces were promising and are in line with outcomes of earlier studies that used SVM classifiers. Quantitative comparison of extracted vegetation heights with reference trees showed moderate vertical reliability. Due to limitations of photogrammetric DSM products similar patterns in the geographic location of object height outliers were detected in both study areas. Outliers occurred in shadowed areas, on water surfaces and in forests with high or extremely high tree stands. Despite their limitations in high vertical accuracy, Pléiades (tri-)stereo products can be effectively used for vegetation height stratification where the application does not require the accuracy of laser scanning. The elaborated methodology is reliable in discriminating main land cover categories and promising in estimating height of vertically relatively homogenous vegetation. Further development in reducing manual interference in the segmentation and classification phases is foreseen. Results of this work will practically support a settlement scale quality of urban life analysis in Szeged, and second the workflow will form methodological input for a green monitoring and evaluation strategy in Salzburg.
